# Escore de Cálcio Coronário e Estratificação do Risco de Doença Arterial Coronariana em Pacientes com Acidente Vascular Encefálico Isquêmico Aterosclerótico e não-Aterosclerótico

**DOI:** 10.36660/abc.20190616

**Published:** 2020-12-01

**Authors:** Edson Marcio Negrão, Maria Cristina Del Negro Barroso Freitas, Patricia Beatriz Christino Marinho, Thiago Falcão Hora, Vinicius Viana Abreu Montanaro, Bernardo Jose Alves Ferreira Martins, Sergio Henrique Rodolpho Ramalho

**Affiliations:** 1 Rede SARAH de Hospitais de Reabilitação BrasíliaDF Brasil Rede SARAH de Hospitais de Reabilitação - Clínica Médica, Brasília, DF - Brasil; 2 Rede SARAH de Hospitais de Reabilitação BrasíliaDF Brasil Rede SARAH de Hospitais de Reabilitação – Neurologia, Brasília, DF - Brasil; 3 Rede SARAH de Hospitais de Reabilitação BrasíliaDF Brasil Rede SARAH de Hospitais de Reabilitação – Radiologia, Brasília, DF – Brasil

**Keywords:** Acidente Vascular Encefalico, Doença da Artéria Coronariana, Sinalização do Cálcio, Dislipidemias, Hipertensão, Diabetes Mellitus

## Abstract

**Fundamento:**

O acidente vascular encefálico isquêmico (AVEi) e a doença arterial coronariana (DAC) coexistem frequentemente e compartilham fatores de risco para doença aterosclerótica. Segundo a *American Heart Association* , os subtipos de AVEi podem ser considerados equivalentes de risco para DAC, mas a evidência para o AVEi não-aterosclerótico não está bem definida. Além disso, o escore de cálcio coronário (CAC) é um marcador preciso para estimar o risco de DAC. Entretanto, a distribuição do CAC pelos subtipos de AVEi ainda não foi bem caracterizada.

**Objetivos:**

Comparar o CAC entre os grupos de AVEi ateroscleróticos e não ateroscleróticos, e determinar quais covariáveis estão associadas a CAC alto no AVEi

**Métodos:**

Em um estudo transversal, incluímos todos os pacientes com AVEi, com idades entre 45 a 70 anos no momento do acidente vascular, consecutivamente admitidos em um hospital de reabilitação entre agosto de 2014 e dezembro de 2016, sem DAC prevalente. Todos os pacientes passaram por tomografia computadorizada (TC), para medir o CAC. CAC≥100 foi considerado alto risco de DAC. O nível de significância foi p<0,05.

**Resultados:**

Dos 244 pacientes estudados (média de idade de 58,4±6,8 anos; 49% do sexo feminino), 164 (67%) apresentavam etiologia não-aterosclerótica. As proporções de CAC≥100 foram semelhantes entre os grupos ateroscleróticos e não-ateroscleróticos (33% [n=26] x 29% [n=47]; p= 0,54). Entre todos os pacientes com AVEi, apenas os de idade ≥60 anos foram associados independentemente a CAC≥100 (RC 3,5; 95% IC 1,7-7,1), ajustado para hipertensão, dislipidemia, diabetes, sedentarismo, e histórico familiar de DAC.

**Conclusão:**

O AVEi aterosclerótico não apresentou risco maior de DAC quando comparado ao AVEi não-aterosclerótico de acordo com o CAC. Apenas a faixa etária ≥60 anos – mas não a etiologia - foi associada independentemente a CAC≥100. (Arq Bras Cardiol. 2020; 115(6):1144-1151)

## Introdução

O acidente vascular encefálico isquêmico (AVEi) e a doença arterial coronariana (DAC) são as principais causas de mortalidade em todo o mundo.^1^ A prevalência simultânea estimada das duas doenças pode chegar a 70%, com qualquer grau de DAC.^2^ Além disso, o risco absoluto de infarto do miocárdio é 2,2% por ano em pacientes que tiveram AVEi ou ataque isquêmico transitório,^3^ e o risco de eventos cardíacos fatais é aproximadamente duas vezes o risco de acidente vascular fatal após 5 anos de sobrevivência ao AVEi.^4^

A íntima associação entre AVEi e DAC pode ser explicada pelo fato de as duas doenças terem fisiopatologias e fatores de risco para aterosclerose semelhantes, como hipertensão arterial sistêmica, dislipidemia e tabagismo, compartilhando objetivos preventivos e terapêuticos comuns. Segundo a *American Heart Association* e a *American Stroke Association* , os subtipos do AVEi podem ser considerados equivalentes a DAC, mas a evidência para AVI não-aterosclerótico não está bem definida.^5^

A aterosclerose de grandes artérias é uma etiologia frequente do AVEi, variando entre 9% e 24% de todos os casos de AVEi alternando com os subtipos de doenças de pequenos vasos e cardioembólicas,^6 , 7^ dependendo das características da coorte e distribuição de fatores de risco.^8 , 9^ Entretanto, não se estabeleceu definitivamente se os subtipos não-ateroscleróticos de AVEi apresentam o mesmo nível de risco de DAC que o AVEi aterosclerótico. Além disso, a aterosclerose coronária não diagnosticada em pacientes com AVEi varia em prevalência e gravidade. Estenose coronária angiográfica maior que 50% é encontrada em 26% dos pacientes com AVEi e sem histórico conhecido de DAC.^10^ Como alternativa, utilizando o escore de cálcio coronário (CAC) como estratégia de estratificação de risco não invasiva, a prevalência de DAC em AVEi pode chegar a 70% dos pacientes, nos quais um quarto está sob risco muito alto (CAC>400).^11^

Portanto, nosso objetivo foi comparar o CAC entre AVEi aterosclerótico e não-aterosclerótico, como marcador de risco de DAC. Além disso, determinamos quais covariáveis estão associadas a CAC alto no AVEi, além da etiologia. Aventamos a hipótese de que o cálcio coronário seria maior em AVEi aterosclerótico do que em AVEi de outras etiologias, servindo como ferramenta valiosa de triagem para estratificação de risco no AVEi.

## Métodos

Em um desenho transversal, incluímos todos os pacientes com diagnóstico de AVEi, com idades entre 45 a 70 anos no momento do evento neurológico, que foram consecutivamente admitidos na Unidade de Brasília da Rede SARAH de Hospitais de Reabilitação entre agosto de 2014 e dezembro de 2016. Excluímos pacientes com diagnósticos anteriores de DAC, considerando que nossa população alvo eram indivíduos em risco e sem doença estabelecida. Todos os pacientes assinaram o termo de consentimento informado antes de serem incluídos no estudo. Este estudo foi aprovado pelo Comitê de Ética da instituição.

O AVEi foi confirmado por avaliação clínica e por um método de imagem. A etiologia do acidente vascular foi classificada por dois neurologistas independentes usando um sistema informatizado baseado no *Trial of ORG 10172 in Acute Stroke Treatment* (TOAST - Estudo de ORG 10172 sobre Tratamento de Acidente Vascular Agudo) do *Stop Stroke Study Causative Classification System* (SSS-CCS - Sistema de Classificação de Causas do Estudo sobre Interrupção de Acidente Vascular) disponível *on-line* .^12 , 13^ As discordâncias foram resolvidas por um terceiro neurologista independente. Para essa análise, todas as etiologias não ateroscleróticas foram atribuídas a um único grupo para regressão logística.

A investigação etiológica incluiu ecocardiograma transtorácico, radiografia do tórax, eletrocardiograma, neuroimagem (RNM ou TC), estudos vasculares intracranianos não invasivos (angiografia por ressonância magnética, angiografia por tomografia computadorizada, e Doppler transcraniano). Quando necessário, foram realizados ecocardiograma transesofágico e monitoramento por Holter de 24 horas. Outros exames também foram solicitados na avaliação clínica, tais como, hemograma completo, função renal, exames de doenças endêmicas (HIV, sífilis e doença de Chagas). Em alguns pacientes, também investigamos trombofilia (antitrombina III, deficiência de proteínas C e S, pesquisa de síndrome antifosfolipídica, mutações de protrombina e do fator V de Leiden, e níveis de homocisteína).

### Escore de cálcio coronário

Todos os pacientes foram submetidos a determinação de CAC. Realizamos uma aquisição de imagem axial do coração com cortes de 3 mm em tomografia computadorizada em multidetector, sincronizada com o eletrocardiograma. Três modelos de tomógrafos foram usados: Siemens Sensation 64, Siemens Perspective 128, e Siemens Definition. As imagens foram analisadas no software Siemens Syngo Calcium Scoring, e os radiologistas desconheciam a etiologia do acidente vascular. Análises semiautomáticas de placas calcificadas foram realizadas com imagens eletrônicas identificadas com mais de 3 pixels adjacentes com densidade acima de 130 unidades Hounsfield.^14^ O alto risco foi definido como um CAC≥100, considerado como um valor de corte validado em termos de prognóstico.^15^ Como análise de sensibilidade, também comparamos a distribuição do risco mais baixo, definido como CAC=0 entre ambos os grupos de AVEi.

### Caracterização das variáveis estudadas

As variáveis do estudo foram definidas conforme apresentado abaixo:

Hipertensão arterial sistêmica: pressão arterial sistólica maior que 140 mmHg, pressão arterial diastólica maior que 90 mmHg; uso de medicamento anti-hipertensivo.

Dislipidemia: LDL acima de 160 mg/dL ou uso de fármaco hipolipemiante.

Diabetes mellitus: glicemia em jejum acima de 126 mg/dL ou uso de fármaco hipoglicemiante e/ou insulina.

Sedentarismo: menos de 150 minutos de exercício moderado por semana.

Obesidade: índice de massa corporal acima de 30 kg/m^2^

Histórico familiar de DAC prematura: parentes de primeiro grau diagnosticados com DAC com < 50 anos, para homens e < 65 anos para mulheres.

Tabagismo: uso de cigarros autorrelatado por no mínimo um ano ou ser ex-fumante por menos de cinco anos.

Escala de Rankin modificada: utilizada para medir o grau de incapacidade ou dependência de outros para realizar atividades diárias. Foi calculada por um neurologista na entrada do programa de reabilitação.^18^

Estimativa de risco de doença cardiovascular aterosclerótica (ASCVD, do inglês *Atherosclerotic Cardiovascular Disease* ) em 10 anos: utilizamos equações de coorte agrupadas para estimar o risco de eventos coronarianos em 10 anos, classificados em: risco baixo (<5%), risco limítrofe (5-7,4%), risco intermediário (7,5-19,9%), risco alto (≥ 20%).^19^

### Análise estatística

Variáveis categóricas foram apresentadas como número absoluto e proporção ou como variáveis contínuas como média ± DP ou mediana (25-75° percentil). O teste de normalidade Kolmogorov-Smirnov foi utilizado para verificar a distribuição. Para atender ao objetivo principal, grupos ateroscleróticos e não-ateroscleróticos foram comparados utilizando o teste qui-quadrado para variáveis categóricas, e *t* para amostras independentes ou teste U Mann-Whitney, conforme apropriado, para variáveis contínuas.

Para atender a nosso objetivo secundário, utilizamos um modelo de regressão logística multivariada para investigar as covariáveis associadas a risco mais alto de DAC, representada como CAC≥100. A variável dependente foi CAC dicotomizado entre ≥100 e <100. As covariáveis candidatas a serem testadas como independentes no modelo final foram consideradas com base em evidência clínica, informações disponíveis na literatura e análise univariada, e, nesse caso, o critério de decisão foi um p valor < 0,20. Portanto, o modelo multivariado final incluiu idade > 60 anos, hipertensão, dislipidemia, diabetes, sedentarismo e histórico familiar de DAC prematura. O nível de significância aceito geral foi p < 0,05. As análises foram conduzidas em SPSS 20.

## Resultados

De um total de 269 pacientes aptos, 25 não compareceram às avaliações adicionais, resultando em uma amostra final de 244 pacientes para análise. Não houve suspeita de infarto do miocárdio conforme análise do histórico, eletrocardiograma e ecocardiograma. A frequência do grupo aterosclerótico foi 33% (n=80), sem diferença etária significativa em comparação com o grupo não-aterosclerótico ( Tabela 1 ), que também foram admitidos mais tardiamente para reabilitação. A distribuição de gênero entre os grupos também foi semelhante (49% de mulheres em ambos). Considerando os principais fatores de risco cardiovascular, não se encontrou diferença na frequência de hipertensão, dislipidemia, diabetes, sedentarismo e obesidade. Por outro lado, a frequência de tabagismo e histórico familiar de DAC prematura foram mais altas no grupo aterosclerótico. Embora o escore de ASCVD tenha sido mais alto para o AVEi aterosclerótico, a ASCVD mediana para cada grupo permaneceu entre >7,5% e <20%, portanto ambos foram classificados como risco intermediário. Um CAC mediano maior foi observado em pacientes com AVEi aterosclerótico, apesar de não ter havido diferença estatística em comparação com os pacientes com AVEi não-aterosclerótico.

**Tabela 1:** – Características clínicas da amostra do estudo

	Geral	Não aterosclerótico	Aterosclerótico	p valor
	(n= 244)	(n= 164; 67%)	(n= 80; 33%)	
Idade, anos; média±DP	58,4 ± 6,8	57,8 ± 6,7	59,5 ± 7,0	0,078
Tempo desde o acidente vascular, meses*; média [25-75º percentil]	5,0[3,0-9,0]	5,0 [2,5-8,0]	6,0 [4,0-10,5]	0,019
Mulheres, n(%)	120 (49,2)	81 (49,4)	39 (48,8)	0,925
Hipertensão, n(%)	177 (72,5)	119 (72,6)	58 (72,5)	0,992
Dislipidemia, n(%)	183 (74,9)	123 (75,0)	60 (74,7)	0,833
Tabagismo, n(%)	77 (31,7)	37 (22,7)	40 (50,0)	<0,001
Diabetes, n(%)	69 (28,3)	49 (29,9)	20 (25,0)	0,427
Sedentarismo, n(%)	170 (70,0)	113 (69,0)	57 (71,6)	0,691
Obesidade, n(%)	46 (18,9)	28 (17,1)	18 (22,5)	0,309
Escala Rankin	3,3±0,9	3,3±0,9	3,3±0,9	0,486
Histórico familiar de DAC prematura, n(%)	37 (15,2)	18 (11,3)	19 (23,6)	0,016
Risco cardiovascular (ASCVD) em 10 anos, mediana [25-75º percentil]	9,1 [4,8-15]	8,4 [3,7-13,9]	10,3 [6,2-18,1]	0,013
Escore de cálcio coronário; mediana [25-75º percentil]	9,0 [0,0-129,7]	4,0 [0,0-128,8]	24,6 [(0,0-132,4]	0,510

DAC: doença arterial coronariana; ASCVD: risco de doença cardiovascular aterosclerótica. *Meses desde o acidente vascular até a inclusão no estudo.

**Figura 1:**
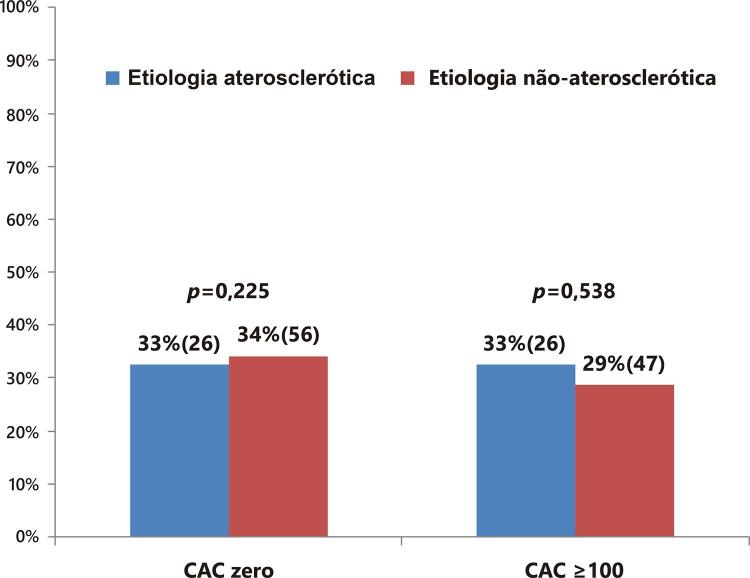
– Prevalência das categorias de escore de cálcio coronário (CAC) em grupos ateroscleróticos e não aterosclerótico.

Para definir a etiologia, 87% dos pacientes passaram por ressonância magnética e 13% fizeram exclusivamente tomografia computadorizada. Os neurologistas discordaram em sete casos (3%), o que exigiu a avaliação de um terceiro neurologista. A etiologia do AVEi aterosclerótico foi a mais prevalente, seguido de 74 (30%) devido a embolia cardioaórtica, 37 (15%) causados por oclusão de pequenas artérias, 14 (6%) devido a outras causas, e 39 (16%) de causas indeterminadas. Como grupo, houve 164 (67%) de casos não-ateroscleróticos. Entre os 80 casos de etiologia aterosclerótica, 18 (23%) se deveram a aterosclerose intracraniana. Os AVEi aterosclerótico e não-aterosclerótico apresentaram proporções similares de pacientes com CAC ≥ 100. Da mesma forma, aqueles com CAC zero também tinham proporções equivalentes entre grupos ( Figura 1 ).

Como a etiologia de AVEi dicotomizado não discriminou CAC ≥ 100, outros possíveis contribuidores foram analisados. Considerando variáveis clinicamente definidas e as estatisticamente diferentes na análise univariada ( tabela 2 ), 6 variáveis entraram no modelo ajustado final: idade (dicotomizada em ≥60 e <60 anos de idade), hipertensão, dislipidemia, tabagismo, diabetes, e histórico familiar de DAC prematura. Ajustando para todas essas covariáveis, apenas a idade ≥60 anos permaneceu independentemente associada a CAC ≥ 100 ( Tabela 3 ).

**Tabela 2:** – Características clínicas e demográficas de pacientes com acidente vascular encefálico isquêmico, pelo ponto de corte de escore de cálcio coronário (CAC) mais alto

	CAC ≥ 100	CAC < 100	p
	n = 72	n =172	
Sexo (feminino)	32 (44)	88 (51)	0,338
Idade ≥60 anos	54 (75)	60 (35)	<0,001
Hipertensão arterial	64 (89)	113(66)	<0,001
Tabagismo	25(36)	52 (32)	0,492
Diabetes	28 (39)	41 (24)	0,017
Dislipidemia	62 (86)	120 (71)	0,008
Sedentarismo	51 (78)	100 (66)	0,72
Obesidade	15 (21)	31 (18)	0,644
Histórico familiar de DAC prematura	14 (23)	20 (12)	0,049

*Os valores são n (%). DAC: doença arterial coronariana.*

**Tabela 3:** – Medidas de associação entre covariáveis clínicas e CAC de alto risco (≥100), em modelo multivariado ajustado final, incluindo todos os pacientes com acidente vascular isquêmico

Variável	OR	95% IC	p
Idade ≥60 anos	3,52	1,72 - 7,18	0,001
Hipertensão arterial	2,35	0,8 - 6,88	0,12
Dislipidemia	1,67	0,7 - 3,98	0,244
Diabetes mellitus	1,15	0,57 - 2,33	0,692
Sedentarismo	1,46	0,68 - 3,14	0,331
Histórico familiar de DAC prematura	1,69	0,73 - 3,88	0,219

*DAC: doença arterial coronariana.*

## Discussão

Os resultados do estudo demonstraram que um terço dos pacientes com AVEi apresentaram etiologia aterosclerótica, seguida de perto por embolia cardioaórtica. Identificou-se que o escore de cálcio coronário foi distribuído similarmente entre AVEi aterosclerótico e não-aterosclerótico, considerando que não foram observadas diferenças clínicas ou estatísticas no escore de Agatston ou na proporção de pacientes com risco mais alto de DAC estimado por um CAC≥100. Entre outros possíveis contribuidores, apenas o tabagismo atual e o histórico familiar de DAC prematura puderam diferenciar a etiologia de AVEi aterosclerótica da não-aterosclerótica - com uma frequência aproximadamente duas vezes mais alta para ambas as características dentre aqueles com AVEi aterosclerótico.

Embora o risco estimado no AVEi aterosclerótico pelo ASCVD tenha sido maior para o grupo AVEi aterosclerótico, em comparação com AVEi não-aterosclerótico, ambos foram classificados no estrato de risco intermediário. Considerando que a equação de ASCVD possivelmente superestima o risco, o escore de CAC poderia melhorar a estratificação do risco individual.^20^

Diferentemente de nossa hipótese, o risco de acordo com a estratificação pelo CAC foi semelhante entre os grupos de AVEi aterosclerótico e não-aterosclerótico. A proporção (aproximadamente um terço) de pacientes com alto risco de DAC (CAC≥100) foi semelhante para ambos os grupos. É interessante observar que essa constatação também é verdadeira entre pacientes com o risco de DAC mais baixo (CAC zero), distribuído similarmente entre os grupos de AVEi. Considerando que as categorias de CAC não puderam distinguir as etiologias de AVEi, tentamos identificar outros possíveis contribuidores associados ao CAC≥100. Depois de considerar as covariáveis clinicamente relevantes, apenas pacientes com 60 anos ou mais tiverem uma probabilidade maior de ter CAC ≥ 100 (OR 3,52; 95% IC 1,72-7,18). A idade é um conhecido fator de risco de DAC, e sua associação com o aumento do CAC está de acordo com outros autores que demonstraram esta correlação em coortes maiores.^21^

O CAC é um marcador bem definido de DAC, que revela com precisão - com uma dose baixa de radiação - a carga aterosclerótica em artérias coronárias,^24^ e tem um valor prognóstico robusto.^25^ O aumento de CAC absoluto é diretamente proporcional à incidencia de eventos coronarianos.^25 , 26^ Dada uma certa variação no escore de CAC absoluto, considerando coortes diferentes, e uma distribuição não normal, a classificação de pacientes em estratos melhora a possibilidade de generalização e aplicação clínica.^17 , 27^ Portanto, o CAC≥100 unidades Agatston é associado a um risco significativamente mais alto de DAC,^15^ enquanto o CAC zero prevê um risco muito baixo de CAC no longo prazo.^26^ Conforme mostramos, o CAC mantém sua habilidade de avaliar o risco cardiovascular individual em pacientes de acidente vascular independentemente de a etiologia do AVEi ser aterosclerótico ou não.

Em relação às características clínicas comuns entre AVEi e DAC, esperávamos que o grupo AVEi aterosclerótico tivesse um risco mais alto de DAC. Entretanto, essa hipótese não foi confirmada. O perfil de DAC semelhante entre os grupos de AVEi aterosclerótico e não-aterosclerótico pode ser atribuído à frequência muito alta - em ambos os grupos etiológicos - de fatores de risco tradicionais de doenças vasculares ateroscleróticas: ≥70% para hipertensão arterial, dislipidemia e sedentarismo. Além disso, a frequência de tabagismo e diabetes em nossa amostra (32% e 28%, respectivamente) foram mais altos do que a prevalência observada na população brasileira: 15% para tabagismo e 9% para diabetes.^28^ Essas constatações e a média de idade relativamente baixa deste estudo podem refletir o mau controle de fatores de risco modificáveis indistintamente presentes em sobreviventes de acidentes vasculares, independentemente da etiologia.

Enfatizando a relação íntima entre DAC e AVEi, Rivera et al. mostraram que, em estudos em autópsias, as placas coronárias estavam presentes em 72% dos pacientes que sofreram acidente vascular fatal, nos quais aproximadamente 27% apresentavam evidência de infarto do miocárdio silencioso. É interessante observar que aterosclerose coronária e infarto do miocárdio foram prevalentes independentemente do subtipo de acidente vascular.^29^

A relação entre aterosclerose extracraniana e DAC é bem estabelecida.^30^ Entretanto, essa associação com aterosclerose intracraniana é controversa,^31^ e parece ser menos frequentemente associada com o AVEi,^32^ pelo menos na população brasileira. Sabe-se que a aterosclerose intracraniana é mais prevalente na população asiática,^32^ mas já se descreveu uma prevalência que pode chegar a até 50% entre a população afroamericana do sexo masculino.^33^ Observamos aterosclerose intracraniana em 23% dos casos de AVI. Em nosso estudo, utilizamos o algoritmo SSS-CCS, que inclui doença aterosclerótica intracraniana e extracraniana no mesmo grupo aterosclerótico. Portanto, pode ter sido menos restritivo, assim como menos discriminativo para a associação que desejamos definir.

A baixa frequência de acidente vascular criptogênico pode ser atribuída à alta qualidade de investigação e ao uso do algoritmo SSS-CCS que padronizou a classificação etiológica, o que também levou a um baixo índice de discordância entre neurologistas. Mesmo com a exclusão de pacientes com DAC prévia, o índice de 30% de acidente vascular causado por embolia cardioaórtica se deve em parte à presença de 11% de pacientes com cardiomiopatia chagásica. A doença de Chagas é um problema clínico comum na América Latina, em que os principais mecanismos do acidente vascular são embolia, devido à presença de aneurisma em ápice do ventrículo esquerdo, disfunção sistólica grave e fibrilação atrial.^34^

Este estudo tem várias limitações. Primeiramente, considerando que nosso hospital é um centro de reabilitação, os critérios de admissão podem influenciar de alguma forma a estimativa geral de frequência de AVEi. Alguns pacientes com admissão tardia podem ter uma precisão diagnóstica limitada da etiologia de AVEi. Pacientes com acidente vascular lacunar foram menos prevalentes do que na literatura, que pode ser explicado por demandas de reabilitação geralmente mais baixas nesse subgrupo. Por outro lado, pacientes com limitações neurológicas graves com um potencial mais restrito para reabilitação são admitidos com menos frequência, e, por motivos semelhantes, pacientes clinicamente instáveis (tratando uma infecção corrente; com demandas cirúrgicas; com problemas endócrino-metabólicos descompensados) não foram admitidos para reabilitação. Embora essa restrição pudesse ter selecionado aterosclerose coronária menos grave, esse foi um critério de inclusão comum para ambos os grupos. Segundo, este é um estudo realizado em um único centro e o tamanho da amostra é relativamente pequeno, mas a prevalência de CAC≥100 entre sobreviventes de AVEi é consistente com os relatos de outros autores (30-45%).^35 , 36^ Terceiro, esperava-se uma proporção de CAC≥100 15 pontos percentuais mais baixa no grupo de AVEi não-aterosclerótico com base em uma observação clínica arbitrária, em concordância com nossa hipótese. Entretanto, na conclusão do estudo, observa-se uma diferença de 4 pontos percentuais ( Figura 1 ), o que pode ter limitado o poder para detectar diferenças entre grupos em relação a nossa pergunta principal.

O ponto forte deste trabalho é a disponibilização de informações sobre o risco de DAC de acordo com o CAC em sobreviventes de acidente vascular em uma população brasileira, e, especialmente, no grupo de AVEi não-aterosclerótico, para os quais as evidências são menos frequentes. De acordo com a *American Heart Association* e a *American Stroke Association* , a população com AVEi não-aterosclerótico deve ser considerada de risco alto para DAC, e estratégias preventivas devem ser consideradas apropriadamente. Entretanto, os acidentes vasculares são mais heterogêneos do que as DAC, especialmente dentro dos subtipos de AVEi não-ateroscleróticos, nos quais os fatores de risco e os resultados associados não são tão bem determinados.^5^ Etiologias não associadas ao alto risco de DAC, tais como forame oval patente e dissecção da artéria vertebral, mais prevalentes em pacientes mais jovens, podem não ser adequadamente representadas nos grupos de AVEi não-aterosclerótico, o que pode ter ocorrido em nossa amostra também. Considerando-se o nível mais baixo de evidência que o AVEi não-aterosclerótico seja equivalente ao risco de DAC, ainda é necessário ter validação prognóstica, e, portanto, a generalização deve ser interpretada com cuidado. Apresentamos dados sobre essa lacuna, demonstrando que o CAC pode ser utilizado para estimar risco individual de DAC em AVEi, mostrando perfis de risco semelhantes entre os subtipos aterosclerótico e não-aterosclerótico, pelo menos em nossa população, considerando a alta frequência dos fatores de risco de DCV. É importante observar que, ainda que o CAC não tenha conseguido discriminar as etiologias de AVEi em nossa análise, ele melhora a estratificação de risco individual para DAC na população geral,^32^ mesmo em pacientes de alto risco,^37^ cuja aplicabilidade parece ter sido preservada para pacientes com AVEi, independentemente da etiologia.

## Conclusões

Na população estudada, o acidente vascular encefálico isquêmico de etiologia aterosclerótica não apresentou maior risco de DAC se comparado ao acidente vascular encefálico isquêmico não-aterosclerótico de acordo com o CAC. Idade maior ou igual a 60 anos foi a única variável independente associada ao CAC ≥ 100. Em sobreviventes de acidente vascular encefálico isquêmico, o CAC deve ser considerado para a estratificação de risco individual de DAC, mesmo em etiologias não- ateroscleróticas.
