# Supplementary material for: Escore de Cálcio Coronário e Estratificação do Risco de Doença Arterial Coronariana em Pacientes com Acidente Vascular Encefálico Isquêmico Aterosclerótico e não-Aterosclerótico
Source: Arq Bras Cardiol. 2020 Dec 1;115(6):1144–51. [Article in Portuguese] doi: 10.36660/abc.20190616 (PMC8133727; doi:10.36660/abc.20190616)
Supplement: Translation

# Translation: Coronary Calcium Score and Stratification of Coronary Artery Disease Risk in Patients with Atherosclerotic and Non-Atherosclerotic Ischemic Stroke

**Authors:** Edson Marcio Negrão, Maria Cristina Del Negro Barroso Freitas, Patricia Beatriz Christino Marinho, Thiago Falcão Hora, Vinicius Viana Abreu Montanaro, Bernardo Jose Alves Ferreira Martins

**Affiliations:** 1 Brazil Rede SARAH de Hospitais de Reabilitação - Clínica Médica, Brasília, DF - Brazil; 2 Brazil Rede SARAH de Hospitais de Reabilitação – Neurologia, Brasília, DF - Brazil; 3 Brazil Rede SARAH de Hospitais de Reabilitação – Radiologia, Brasília, DF – Brazil

**Keywords:** Stroke, Coronary Artery Disease, Calcium Signaling, Dyslipidemias, Hypertension, Diabetes Mellitus

## Abstract

**Background:**

Ischemic Stroke (IS) and Coronary Artery Disease (CAD) frequently coexist and share atherosclerotic disease risk factors. According to the American Heart Association, IS subtypes may be considered CAD risk equivalents, but the evidence for non-atherosclerotic IS is uncertain. Additionally, the Coronary Calcium Score (CCS) is an accurate marker to address CAD risk; however, CCS distribution between IS subtypes is not well characterized.

**Objectives:**

To compare the CCS between atherosclerotic and non-atherosclerotic IS groups; and to determine which covariates were associated with high CCS in IS.

**Methods:**

This cross-sectional design included all patients with IS, 45 to 70 years of age at the time of the stroke, consecutively admitted to a rehabilitation hospital between August 2014 and December 2016, without prevalent CAD. All patients underwent CT scanning for CCS measurement. CCS≥100 was considered a high risk for CAD, with a significance level of p<0.05.

**Results:**

From the 244 studied patients (mean age 58.4±6.8 years; 49% female), 164 (67%) had non-atherosclerotic etiology. The proportions of CCS≥100 were similar between the atherosclerotic and the non-atherosclerotic groups (33% [n=26] x 29% [n=47]; p= 0.54). Among all IS patients, only age ≥60 years was independently associated with CCS≥100 (OR 3.5; 95%CI 1.7-7.1), accounting for hypertension, dyslipidemia, diabetes, sedentarism, and family history of CAD.

**Conclusion:**

Atherosclerotic IS did not present a greater risk of CAD when compared to non-atherosclerotic IS according to CCS. Only age ≥60 years, but not etiology, was independently associated with CCS≥100.

## Introduction

Ischemic Stroke (IS) and Coronary Artery Disease (CAD) are the leading causes of mortality worldwide.^1^The estimated simultaneous prevalence of both diseases could be as high as 70%, with any degree of CAD.^2^Additionally, the absolute risk of myocardial infarction is 2.2% per year in patients who had IS or transient ischemic attack,^3^and the risk of fatal cardiac events is approximately twice the risk of recurrent fatal stroke at 5 years after surviving stroke.^4^

This close relationship between IS and CAD may be explained by similar pathophysiology and risk factors for atherosclerosis in both diseases, like systemic arterial hypertension, dyslipidemia, and smoking, which share preventive and therapeutic goals. According to the American Heart Association and the American Stroke Association, IS subtypes may be considered CAD risk equivalents, but the evidence for non-atherosclerotic IS is uncertain.^5^

Large artery atherosclerosis is a frequent IS etiology, ranging from 9% to 24% of overall IS cases, alternating with cardioembolic and small vessel disease subtypes as the most prevalent IS causes,^6 , 7^depending on the cohort characteristics and risk factor distribution.^8 , 9^However, it is not well established whether non-atherosclerotic subtypes of IS are under the same level of CAD risk as atherosclerotic IS. Additionally, undiagnosed coronary atherosclerosis in IS patients varies in prevalence and severity. Angiographic coronary stenosis of greater than 50% is present in 26% of the patients with IS and no known history of CAD.^10^Alternatively, using the coronary calcium score (CCS) as a non-invasive risk stratification strategy, the prevalence of CAD in IS can be as high as 70%, in whom approximately one quarter are under a very high risk (CCS>400).^11^

Therefore, this study aimed to cross-sectionally compare the CCS between atherosclerotic and non-atherosclerotic IS, as a marker of CAD risk. In addition, this study determined which covariates were associated with high CCS in IS, other then etiology. We therefore hypothesized that coronary calcium would be higher in atherosclerotic IS than in IS from other etiologies, serving as a valuable screening tool for risk stratification in IS.

## Methods

This cross-sectional design included all patients with a diagnosis of IS, 45 to 70 years of age, at the time of the neurologic event, admitted consecutively at Brasília Unit of the Sarah Network of Rehabilitation Hospitals between August 2014 and December 2016. Patients with previous diagnosis of CAD were excluded, given that our target population was at-risk individuals and not with established disease. All patients signed the informed consent form prior to study enrollment. This study was approved by the institution’s Ethics Committee.

IS was confirmed by clinical evaluation and an image method. Stroke etiology was classified by two independent neurologists, using a computerized system based on the Stop Stroke Study Causative Classification System (SSS-CCS) Trial of ORG 10172 in Acute Stroke Treatment (TOAST) available on line.^12 , 13^Disagreements were resolved by a third independent neurologist. For this analysis, all non-atherosclerotic etiologies were adjudicated in one group for logistic regression.

Etiological investigation included transthoracic echocardiography, chest radiograph, EKG, neuroimaging (MRI or CT), non-invasive intracranial vascular studies (magnetic resonance angiography, computed tomography angiography, and transcranial Doppler). If necessary, transesophageal echocardiography and 24-hour Holter monitoring were performed. Other exams were also requested upon clinical evaluation, such as complete blood count, renal function, screening for endemic diseases (HIV, syphilis, and Chagas’ disease). In selected patients, thrombophilia (antithrombin III, protein C and S deficiency, search for antiphospholipid syndrome, prothrombin and factor V of Leiden mutations and homocysteine levels) was also investigated.

### Coronary calcium score

All patients underwent CCS determination. A prospective axial image of the heart was acquired using multidetector computed tomography cuts of 3mm, synchronized with the EKG. Three models of CT scanners were used: Siemens Sensation 64, Siemens Perspective 128, and Siemens Definition. The images were analyzed in the Siemens Syngo Calcium Scoring software and the radiologists were blinded to the stroke etiology. Semiautomatic analysis of calcified plaques was performed with electronic identified images with more than 3 adjacent pixels with density greater than 130 Hounsfield Units.^14^High risk was defined as a CCS≥100, considered as a prognostically validated cut-off.^15^As a sensitivity analysis, the distribution of the lowest risk, defined as a CCS=0 between both IS groups, was also compared.

### Characterization of the studied variables

Study variable were defined as follows:

Systemic Arterial hypertension: systolic arterial pressure more than 140 mmHg, diastolic arterial pressure more than 90 mmHg; use of antihypertensive drug.

Dyslipidemia: LDL more than 160 mg/dL or use of lipid-lowering agent.

Diabetes mellitus: fasting blood glucose more than 126 mg/dL or use of hypoglycemic agent and/or insulin.

Sedentary life style: less than 150 minutes of moderate exercise per week.

Obesity: body mass index more than 30 kg/m^2^

Family history of premature CAD: first degree relatives with a diagnosis of CAD of < 50 years old in men and of < 65 years old in women.

Smoking: self-reported current use of cigarettes for at least one year or cessation of smoking for less than five years.

Modified Rankin Scale: used to measure the degree of disability or dependence in one’s daily activities. This was calculated by one neurologist upon rehabilitation program admission.^18^

10 year atherosclerosis cardiovascular disease (ASCVD) estimation risk: the pooled cohort equations was used to estimate the risk of coronary events in 10 years, classified as: low risk (<5%), borderline risk (5-7.4%), intermediate risk (7.5-19.9%), high risk (≥ 20%).^19^

### Statistical analysis

Categorical variables are presented as count with proportion or as continuous variables as mean ± SD or median (25-75^th^percentile). Kolmogorov-Smirnov normality test was used to verify the distribution. To address the main objective, atherosclerotic and non-atherosclerotic groups were compared using the chi-square test for categorical variables, and the independent samples *t* test or Mann-Whitney U test, as appropriate, for continuous variables.

To address our secondary objective, a multivariate logistic regression model was used to investigate the covariates associated with a higher CAD risk, represented as a CCS≥100. The dependent variable was CCS dichotomized between ≥100 and <100. The candidate covariates to be tested as independents in the final model were considered on the basis of clinical evidence, information available in the literature and univariate analysis; in this case, the decision criterion was a p-value < 0.20. Thus, the final multivariate model included age >60years, hypertension, dyslipidemia, diabetes, sedentarism, and family history of premature CAD. The overall accepted level of significance was p < 0.05. Analyses were conducted in SPSS 20.

## Results

From a total of 269 eligible patients, 25 did not attend further evaluations, resulting in a final sample of 244 patients for analysis. No silent myocardial infarction was suspected after enrollment according to patient medical history, EKG, and echocardiography. The atherosclerotic group frequency was 33% (n=80), without a significant age difference compared to the non-atherosclerotic group ( Table 1 ), who were also admitted slightly later. Gender distribution between groups was also similar (49% of female gender for both). Considering the main cardiovascular risk factors, no difference was found in the hypertension, dyslipidemia, diabetes, sedentarism, and obesity rates. On the other hand, the rates of smoking and family history of premature CAD were higher in the atherosclerotic group. Although the ASCVD score was higher for atherosclerotic IS, the median ASCVD for each group was >7.5% and <20%; therefore, both were classified as an intermediate risk. A greater median CCS was observed in atherosclerotic IS patients; however, with no statistical difference when compared to non-atherosclerotic IS patients.

**Table 1:** – Clinical characteristics of the Study Sample

	Overall	Non-atherosclerotic	Atherosclerotic	p-value
	(n= 244)	(n= 164; 67%)	(n= 80; 33%)	
Age, years; mean±SD	58.4 ± 6.8	57.8 ± 6.7	59.5 ± 7.0	0.078
Time since stroke, months*; median [25-75^th^percentile]	5.0[3.0-9.0]	5.0 [2.5-8.0]	6.0 [4.0-10.5]	0.019
Female, n(%)	120 (49.2)	81 (49.4)	39 (48.8)	0.925
Hypertension, n(%)	177 (72.5)	119 (72.6)	58 (72.5)	0.992
Dyslipidemia, n(%)	183 (74.9)	123 (75.0)	60 (74.7)	0.833
Smoking, n(%)	77 (31.7)	37 (22.7)	40 (50.0)	<0.001
Diabetes, n(%)	69 (28.3)	49 (29.9)	20 (25.0)	0.427
Sedentary lifestyle, n(%)	170 (70.0)	113 (69.0)	57 (71.6)	0.691
Obesity, n(%)	46 (18.9)	28 (17.1)	18 (22.5)	0.309
Rankin scale	3.3±0.9	3.3±0.9	3.3±0.9	0.486
Family history of premature CAD, n(%)	37 (15.2)	18 (11.3)	19 (23.6)	0.016
Current 10-year ASCVD risk; median [25-75^th^percentile]	9.1 [4.8-15]	8.4 [3.7-13.9]	10.3 [6.2-18.1]	0.013
Coronary calcium score; median [25-75^th^percentile]	9.0 [0.0-129.7]	4.0 [0.0-128.8]	24.6 [(0.0-132.4]	0.510

CAD: coronary artery disease; ASCVD: atherosclerotic cardiovascular disease risk. *Months from index stroke to enrollment in the study.

To define the etiology, 87% of the patients underwent magnetic resonance imaging and 13% only a computed tomography. Neurologists disagreed in seven cases (3%), requiring the evaluation of a third neurologist. Atherosclerotic IS etiology was the most prevalent, followed by 74 (30%) due to cardio-aortic embolism, 37 (15%) caused by small artery occlusion, 14 (6%) due to other causes, and 39 (16%) of undetermined causes. As a group, there were 164 (67%) non-atherosclerotic cases. Among the 80 cases of atherosclerotic etiology, 18 (23%) were due to intracranial atherosclerosis. Atherosclerotic and non-atherosclerotic IS showed similar proportions of patients with CCS ≥ 100. Similarly, those with CCS zero also had equivalent proportions between groups ( Figure 1 ).

**Figure 1:** – Prevalence of coronary calcium score (CCS) categories in atherosclerotic and non-atherosclerotic groups.

As dichotomized IS etiology did not discriminate CCS ≥ 100, other potential contributors were analyzed. Considering clinically defined variables and those statistically different in the univariate analysis ( table 2 ), 6 variables entered the final adjusted model: age (dichotomized in ≥60 and <60 years old), hypertension, dyslipidemia, smoking, diabetes, and family history of premature CAD. Accounting for all those covariates, only age ≥60 years remained independently associated with CCS ≥100 ( Table 3 ).

**Table 2:** – Clinical and demographic characteristics from the overall ischemic stroke patients, by the higher coronary calcium score (CCS) cut-off point

	CCS ≥ 100	CCS < 100	p
	n = 72	n = 172	
Sex (female)	32 (44)	88 (51)	0.338
Age ≥60 years	54 (75)	60 (35)	< 0.001
Arterial hypertension	64 (89)	113(66)	< 0.001
Smoking	25(36)	52 (32)	0.492
Diabetes	28 (39)	41 (24)	0.017
Dyslipidemia	62 (86)	120 (71)	0.008
Sedentary lifestyle	51 (78)	100 (66)	0.72
Obesity	15 (21)	31 (18)	0.644
Family history of premature CAD	14 (23)	20 (12)	0.049

*Values are n (%). CAD: coronary artery disease.*

**Table 3:** – Measures of association between clinical covariates and higher risk CCS (≥ 100), in final adjusted multivariate model, from the overall ischemic stroke patients.

Variable	OR	95% CI	p
Age ≥ 60 years	3.52	1.72 - 7.18	0.001
Arterial hypertension	2.35	0.8 - 6.88	0.12
Dyslipidemia	1.67	0.7 - 3.98	0.244
Diabetes mellitus	1.15	0.57 - 2.33	0.692
Sedentary lifestyle	1.46	0.68 - 3.14	0.331
Family history of premature CAD	1.69	0.73 - 3.88	0.219

*CAD: coronary artery disease.*

## Discussion

Our results showed that one third of stroke patients presented atherosclerotic etiology, closely followed by cardio-aortic embolism. We found that the coronary calcium score was similarly distributed between atherosclerotic and non-atherosclerotic IS, given no clinical or statistical differences were observed in the Agatston score or in the proportion of patients within a higher CAD risk, estimated by a CCS≥100. Among other potential contributors, only current smoking and family history of premature CAD could differentiate those with atherosclerotic IS when compared to non-atherosclerotic etiology– with approximately twice higher frequency for both characteristics in atherosclerotic IS.

Although ASCVD estimated risk was greater in the atherosclerotic IS group, as compared to the non-atherosclerotic IS group, both were classified in the intermediate risk stratum. Considering that the ASCVD equation potentially overestimates the risk, CCS could potentially improve the individual risk stratification.^20^

Differently from our hypothesis, the risk according to CCS strata was similar between atherosclerotic and non-atherosclerotic IS. The proportion (approximately one third) of patients with a high CAD risk (CCS≥100) was similar for both groups. Interestingly, this finding was also true amongst patients with the lowest CAD risk (CCS zero), similarly distributed between the IS groups. Given that CCS categories did not distinguish IS etiologies, we tried to identify other potential contributors associated with CCS≥100. After accounting for clinically relevant covariates, only patients with 60 years or more had a higher likelihood of having a CCS ≥ 100 (OR 3.52; 95% CI 1.72-7.18). Age is a well-known risk factor for CAD, and its association with increasing CCS is in agreement with other authors who have demonstrated it in larger cohorts.^21^

CCS is a well-defined marker of CAD, which accurately reveals - with a low dose of radiation - an atherosclerotic burden in coronary arteries,^24^and has a robust prognostic value.^25^An absolute increase in CCS is proportional to coronary event rates.^25 , 26^Given some variation in the absolute CCS score, considering different cohorts, and a non-normal distribution, classifying patients within strata improves generalizability and clinical application.^17 , 27^Therefore, CCS≥100 Agatston units are associated with a significantly higher CAD risk,^15^while CCS of zero predicts a very low long term risk of CAD.^26^As we showed, CCS keeps its ability to assess individual cardiovascular risk in stroke patients regardless of whether the IS etiology is atherosclerotic or not.

Regarding the shared clinical characteristics between IS and CAD, we expected that the atherosclerotic IS group would have a greater risk of CAD. However, our hypothesis was not confirmed. The similar CAD risk profile between the atherosclerotic and non-atherosclerotic IS groups can be attributed to a very high frequency – in both etiological groups – of traditional risk factors for atherosclerotic vascular diseases: ≥70% for arterial hypertension, dyslipidemia, and sedentary lifestyle. Moreover, the smoking and diabetes rates in our sample (32% and 28%, respectively) were higher than the prevalence observed in the Brazilian population: 15% for smoking and 9% for diabetes.^28^These findings and the relatively low mean age in this study, may reflect the poor control of modifiable risk factors indistinctively present in stroke survivors, irrespective of the etiology.

Emphasizing the close relationship between CAD and IS, Rivera et al. showed that, in autopsy studies, coronary plaques were present in 72% of patients with fatal stroke, in whom approximately 27% showed evidence of silent myocardial infarction. Interestingly, coronary atherosclerosis and myocardial infarction were prevalent regardless of the stroke subtypes.^29^

The relationship between extracranial atherosclerosis and CAD is well established.^30^However, this association with intracranial atherosclerosis is controversial^31^and seems to be less frequently associated with IS,^32^at least in the Brazilian population. Intracranial atherosclerosis is known to be more prevalent in the Asian population,^32^but it was described to be as high as 50% among male African Americans as well.^33^We observed intracranial atherosclerosis in 23% of atherosclerotic IS cases. In our study, we used the SSS-CCS algorithm, which includes intracranial and extracranial atherosclerotic disease in the same atherosclerotic etiologic group; therefore, it could have been less restrictive, but also less discriminative for the association we aimed to define.

The low frequency of cryptogenic stroke can be attributed to the high quality of investigation and the use of SSS-CCS algorithm that standardized the etiologic classification, also leading to a low rate of disagreement among neurologists. Even with the exclusion of patients with prior CAD, the rate of 30% of stroke caused by cardio-aortic embolism is in part due to the presence of 11% of patients with Chagas’ cardiomyopathy. Chagas’s disease is a common clinical condition in Latin America, whose main mechanisms for stroke are embolism due to the presence of left ventricular apex aneurysm, severe systolic dysfunction, and atrial fibrillation.^34^

Our study has several limitations. First, considering that our facility is a rehabilitation center, admittance criteria may somehow bias overall IS frequency estimation. Some patients with delayed admittance may have a limited diagnostic precision of IS etiology. Patients with lacunar stroke were less prevalent than in the literature, which could likely be explained by frequently lower rehabilitation demands in this subgroup. In contrast, patients with severe neurologic limitations with a narrow rehabilitation potential are less frequently admitted, and for similar reasons, clinically unstable patients (treating an ongoing infection; with surgical demands; with decompensated endocrine-metabolic conditions) were not admitted for rehabilitation purposes. Although this could have included less severe coronary atherosclerosis, this was a common inclusion criterion for both groups. Second, this is a single center study and the sample size is relatively small, but CCS≥100 prevalence among IS survivors is consistent with other authors’ reports (30-45%).^35 , 36^Third, we expected a CCS≥100 proportion of 15 percentage points lower in the non-atherosclerotic IS group based on an arbitrary clinical observation, which is in agreement with our hypothesis; however, upon concluding the study, a 4 percentage points difference was observed ( Figure 1 ), which could have limited the power to detect between group differences regarding our main question.

The strength of this work is providing information on CAD risk according to CCS in stroke survivors from a Brazilian population and particularly in the non-atherosclerotic IS group, to which evidence is scarcer. According to the American Heart Association and American Stroke Association, the atherosclerotic IS population should be considered a high risk group for CAD, where preventive strategies should be adequately addressed; however, stroke is more heterogeneous than CAD, particularly within the non-atherosclerotic IS subtypes, where traditional risk factors and associated outcomes are less well determined.^5^Etiologies known not to be associated with a high risk for CAD, such as patent foramen ovale and cervical artery dissection, more frequent in younger patients, can be underrepresented in non-atherosclerotic IS groups, and could have been in our sample as well. Given the lower level of evidence to consider non-atherosclerotic IS as CAD risk equivalent, prognostic validation is still necessary; therefore, generalization should be interpreted cautiously. Data on this gap was provided in this study, showing that CCS can be used to address individual CAD risk in IS, showing similar risk profiles between atherosclerotic and non-atherosclerotic subtypes, at least in our population, given the high frequency of traditional CVD risk factors. It is important to note that even though CCS was not able to discriminate IS etiologies in our analysis, it does improve the individual risk stratification for CAD in the general population,^32^even in high-risk patients,^37^whose applicability seems to be preserved for ischemic stroke patients as well, regardless of etiology.

## Conclusions

In the studied population, ischemic stroke of atherosclerotic etiology did not present a greater risk of CAD when compared to non-atherosclerotic ischemic stroke according to CCS. Age equal to or over 60 years was the only variable associated with CCS ≥ 100. In ischemic stroke survivors, CCS should be considered for individual risk stratification for CAD, even in non-atherosclerotic etiologies.
